# Symptom-specific assessment of treatment efficacy: The potential of network estimation techniques

**DOI:** 10.1192/j.eurpsy.2021.145

**Published:** 2021-08-13

**Authors:** L. Boschloo

**Affiliations:** Department Of Clinical, Neuro And Developmental Psychology, Vrije Universiteit Amsterdam / Amsterdam Public Health Research Institute, Amsterdam, Netherlands

## Abstract

**Abstract Body:**

**Introduction:** Most studies on the efficacy of psychiatric treatments consider overall scale scores as outcome measures. A focus on individual symptoms would, however, result in a more precise assessment of treatment efficacy and has potential in improving our understanding of the working mechanisms of treatment. Such an approach may also help in improving the identification of patients who are -based on their pretreatment symptomatology- the most likely to benefit from a particular treatment. **Objectives:** To show the potential of network estimation techniques in a) unraveling the diverse symptom-specific responses to various depression treatments and b) improving the identification of patients who are the most likely to benefit from these treatments. **Methods:** First, we combined patient-level data of multiple trials considering various depression treatments, such as antidepressant medication and (internet-based) cognitive-behavioral therapy. Network estimation techniques were used to determine the complex patterns in which symptom-specific responses to treatment were related. **Results:** Individual clinical symptoms differed substantially in their responses to treatment and these symptom-specific responses were related in a complex manner. Patients suffering from symptoms that were directly affected by a particular treatment were -by definition- the most likely to benefit from that treatment. **Conclusions:** Network estimation techniques were able to unravel the diverse symptom-specific responses to treatment and could help in improving our understanding of the chain of events leading to a clinical response. Information from the networks could also help in improving the identification of patients who were the most likely to benefit from a particular treatment.

**Disclosure:**

No significant relationships.

